# The maxillary incisor labial face tangent: clinical evaluation of maxillary incisor inclination in profile smiling view and idealized aesthetics

**DOI:** 10.1186/s40902-019-0214-4

**Published:** 2019-08-20

**Authors:** Farhad B. Naini, Shaadi Manouchehri, Zaid B. Al-Bitar, Daljit S. Gill, Umberto Garagiola, David Wertheim

**Affiliations:** 1 Kingston and St George’s Hospitals and St George’s Medical School, Blackshaw Road, London, SW17 0QT UK; 2 London, UK; 3 Department of Orthodontics and Pediatric Dentistry, School of Dentistry, University of Jordan, Amman, Jordan; 4 Department of Orthodontics, Great Ormond Street Hospital NHS Foundation Trust and UCLH Eastman Dental Hospital, London, UK; 5 Orthodontics, Maxillofacial and Odontostomatology Unit, School of Dentistry, University of Milan, Milan, Italy; 6 Faculty of Science, Engineering and Computing, Kingston University, London, UK

**Keywords:** Incisor inclination, Smile aesthetics, Maxillary incisor, Proclination, Retroclination

## Abstract

**Background:**

To test the hypothesis that in profile smiling view, for ideal aesthetics, a tangent to the labial face of the maxillary central incisor crowns should be approximately parallel to the true vertical line and thereby perpendicular to the true horizontal line.

**Methods:**

An idealized female image was created with computer software and manipulated using the same software to construct an “ideal” female profile image with proportions, and linear and angular soft tissue measurements, based on currently accepted criteria for idealized Caucasian profiles. The maxillary incisor labial face tangent was altered in 5° increments from 70 to 120°, creating a range of images, shown in random order to 70 observers (56 lay people and 14 clinicians), who ranked the images from the most to the least attractive. The main outcome was the preference ranks of image attractiveness given by the observers.

**Results:**

The most attractive inclination of a tangent to the labial face of the maxillary incisor crowns in profile view in relation to the true horizontal line was 85°, i.e. 5° retroclined from a perpendicular 90° inclination. The most attractive range appears to be between 80 and 90°. Excessive proclination appeared to be less desirable than retroclination. Beyond 105° most observers recommend treatment.

**Conclusion:**

In natural head position, the ideal inclination of the maxillary incisor crown labial face tangent in profile view will be approximately parallel to the true vertical line and thereby approximately perpendicular to the true horizontal line.

## Background

The cornerstone of treatment planning in orthodontics and orthognathic surgery is the relationship between the maxillary incisors and the upper lip, the so-called lip-incisor relationship, and the relationship of this complex to the rest of the face. This relationship depends on the three-dimensional position of the maxillary incisors, i.e. vertically in relation to the degree of incisor exposure, sagittally in terms of incisor protrusion or retrusion, and transversely in terms of the dental midline in relation to the facial midline. Additionally, and importantly, the inclination of the maxillary incisors is imperative in terms of smile aesthetics and occlusal function.

**Fig. 1:**
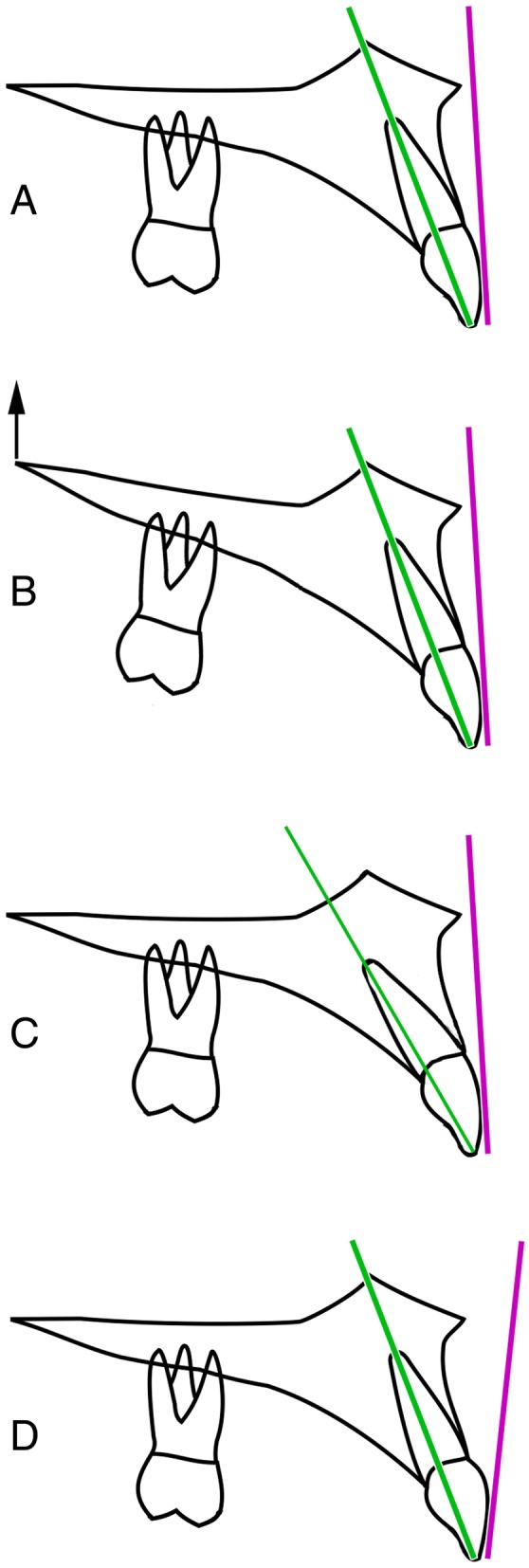
Potential problems with traditional methods of measuring maxillary incisor inclination. **a** The maxillary incisor long axis (line drawn from incisor tip through to the apex) and the labial face tangent, drawn as a tangent to the labial face of the maxillary central incisor in profile view. **b** The inclination of anatomical reference planes is subject to considerable individual variability, e.g. if the maxillary plane is inclined upwards at the back as shown in this diagram, and this would affect the measured incisor long axis inclination in relation to the maxillary plane, but not necessarily the inclination of the maxillary incisor labial face tangent. **c** There may be variation in the incisor crown-root angle, and, as such, the long axis of the incisor tooth may not match that of the crown. **d** There may be considerable variation between the inclination of the long axis of the maxillary central incisor and the inclination of a tangent to the labial face of the tooth crown; the latter is more important from an aesthetic point of view

Traditional measurement of maxillary incisor inclination relies on cephalometric techniques, essentially involving drawing the long axis of the maxillary central incisor from tip to apex and extending the line to meet any of a number of anatomical reference planes, such as the maxillary plane, Frankfort plane or sella-nasion plane, from which incisor inclination is measured. There are a number of potential problems with this approach (Fig. 1). Firstly, the inclination of anatomical reference planes is subject to considerable individual variability [1], and these would affect the measured incisor inclination. Secondly, there may be variation in the incisor crown-root angle, and, as such, the long axis of the incisor tooth may not match that of the crown. Additionally, there is sometimes difficulty in identifying the root apex on a cephalometric radiograph. Finally, and perhaps most significantly, there may be considerable variation between the inclination of the long axis of the maxillary central incisor and the inclination of a tangent to the labial face of the tooth crown, which is far more important from an aesthetic point of view.

Fredericks [2] suggested that the objective of treatment planning should be to achieve a labial crown face inclination parallel to the nasion-pogonion plane. However, this anatomical facial plane is subject to considerable individual variability. More recently, it has been recommended that, for ideal aesthetics, the maxillary incisor crown inclination should be evaluated, clinically in profile smiling view, and cephalometrically, with the patient in natural head position, and that a tangent to the labial face of the central incisor crown should be approximately parallel to the true vertical line, and thereby perpendicular to the true horizontal line [3]. The purpose of this investigation was to test this hypothesis.

## Methods

Ethical approval was provided by the research ethics committee of the University of Jordan, ref.: 5/11/30.

**Fig. 2:**
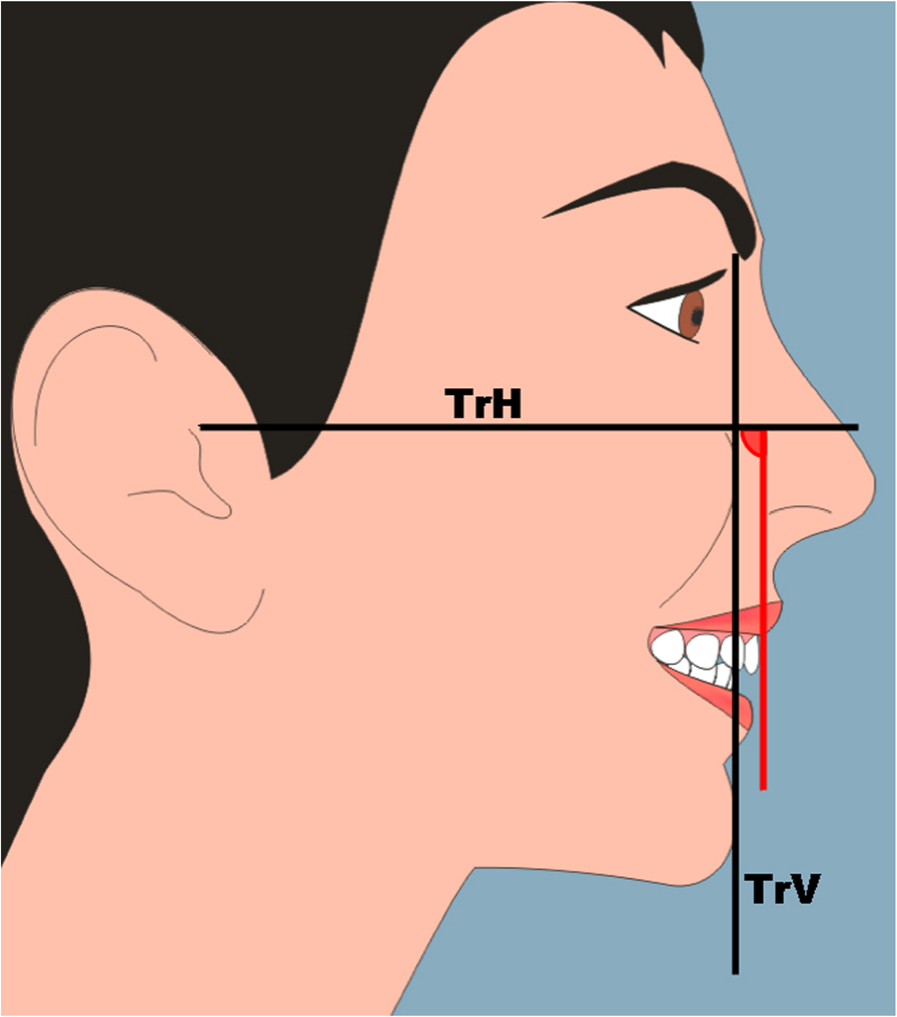
Female smiling profile view; TrH, true horizontal line; TrV, true vertical line; red vertical line is the tangent to the labial face of the maxillary central incisor crown, and the measured angle is the inclination of the labial face tangent in relation to the TrH line

**Fig. 3:**
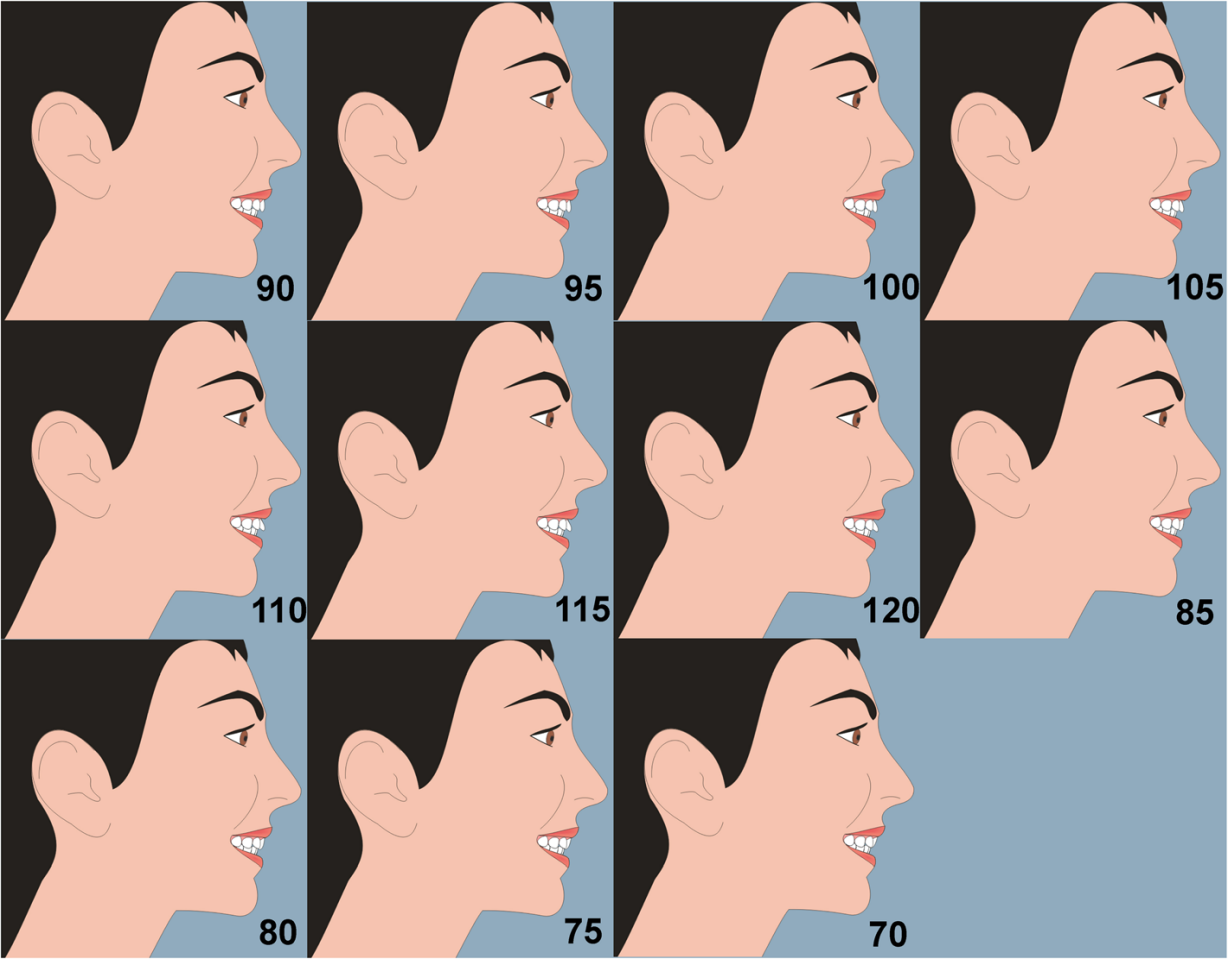
The maxillary incisor labial face tangent was altered in 5° increments from 70 to 120°, creating a range of images. The annotation of the angles of inclination is shown here for ease of reference; these were not on the images shown to the observers

As with profile silhouettes, two-dimensional cartoon-type images have been used to assess the perceptions of facial attractiveness [4–7]. A female image was created with computer software (Adobe® Photoshop® CS2 software). The image was manipulated using the same software to construct an “ideal” female profile image with proportions [3], and linear and angular soft tissue measurements [8–10], based on currently accepted criteria for idealized Caucasian profiles (Fig. 2). The maxillary incisor labial face tangent was altered in 5° increments from 70 to 120°, creating a range of images (Fig. 3). Each image was printed on an A4 sheet of matte photographic paper, and the images were placed in random order on a table top in a room. Based on the results of a pilot study and power calculation, 70 observers took part in the study, separated into two groups (56 lay people, mean age 29 years, age range 17–72 years, male 36%, female 64%, and 14 clinicians, mean age 30 years, age range 23–41 years, male 42%, female 58%). Each observer attended the room unaccompanied and ranked the images from the most to the least attractive. The main outcome was the preference ranks of image attractiveness given by the observers.

### Statistical analysis

Two images in the set had an incisor inclination angle of 105° in order to check assessment repeatability. Software was prepared using MATLAB (The MathWorks Inc., Natick, MA, USA) in order to read the data recorded in spreadsheets and for assessment by descriptive statistics. Graphs were prepared using Minitab v16 (Minitab Inc., USA), and tables prepared using Excel (Microsoft Corporation, USA).

## Results

### Perceived attractiveness of images

Table 1 shows the median, first quartile, and third quartile attractiveness rating from the ordering of images respectively by the clinicians, where 1 indicates the most attractive and 12 indicates the least attractive. Two images were with an incisor inclination of 105° in order to check repeatability of assessment.

**Table 1:** Attractiveness rating of images by clinicians

Inclination (°)	Median	First quartile	Third quartile
70	5	4	6
75	4	2	6
80	2	2	3
85	1	1	1
90	3	3	5
95	6	5	7
100	7	5	7
105	9	8	9
105	8	8	9
110	10	8	10
115	11	11	11
120	12	12	12

**Fig. 4:**
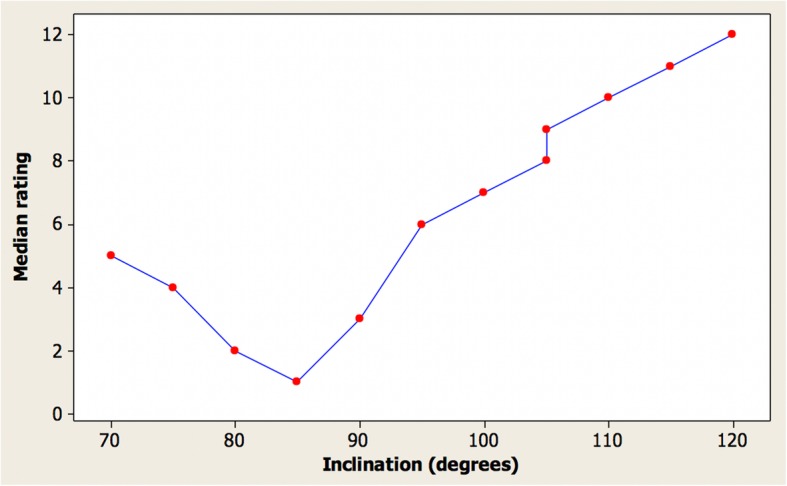
Graph of the median clinician ratings for the female images

The angle which was considered most attractive was 85° for the female images. The first quartile and third quartile values indicate a limited spread in rankings especially for the angles considered least attractive. The repeatability was excellent with almost identical ratings for incisor inclination angles of 105° for the images. Figure 4 shows a graph of the median clinician ratings for the images.

The general trend was that the most attractive images had an incisor inclination of between 80 and 95°. The excellent repeatability can be seen for incisor inclination angles of 105° for the images.

**Fig. 5:**
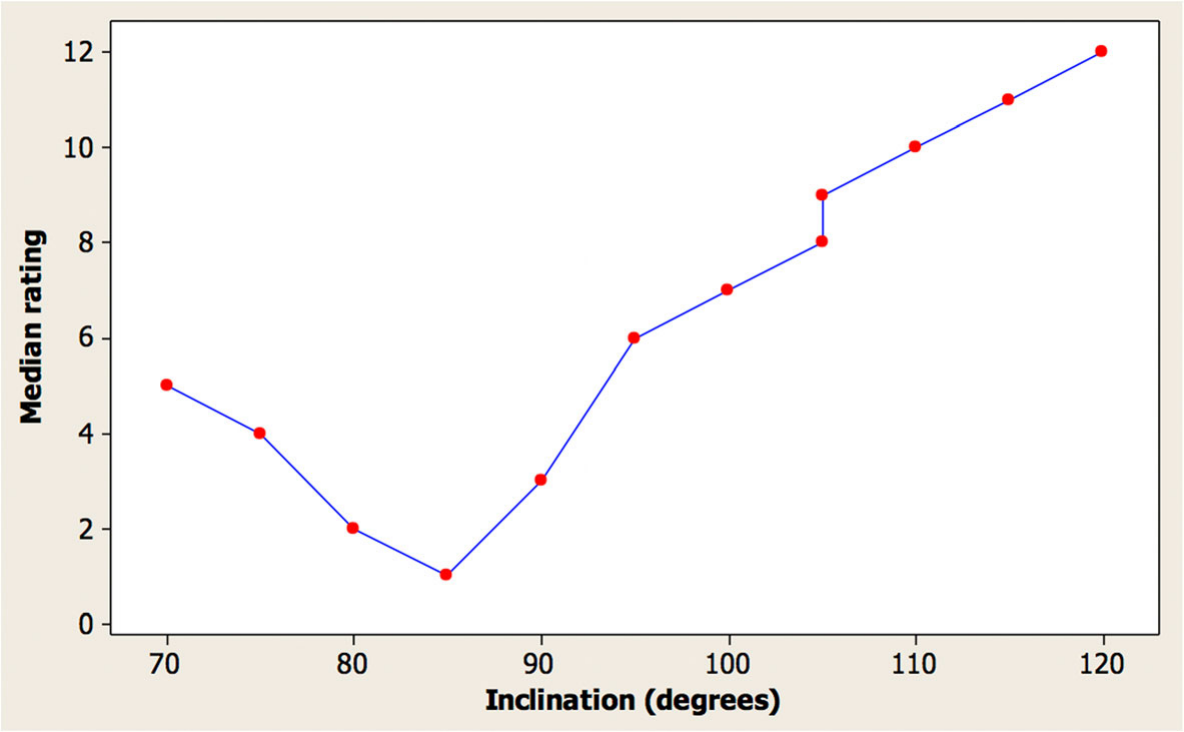
Median rating by laypeople vs. incisor inclination for female images

Table 2 shows the ranking of attractiveness ratings in angle order of the images judged by dentists and laypersons. Overall, the results were similar with an 85° angle being considered as most attractive by both groups. Figure 5 shows the corresponding graph for the laypersons again indicating excellent repeatability for the identical images with an angle of 105°.

**Table 2:** Ranking of images by clinicians compared with laypeople in order of inclination

Inclination (°)	Median (dentists)	Median (laypeople)
70	5	4
75	4	4
80	2	2.5
85	1	1
90	3	3.5
95	6	6
100	7	6
105	9	9
105	8	8
110	10	10
115	11	11
120	12	12

### Proportion expressed as a percentage of each observer group suggesting a desire for treatment

**Fig. 6:**
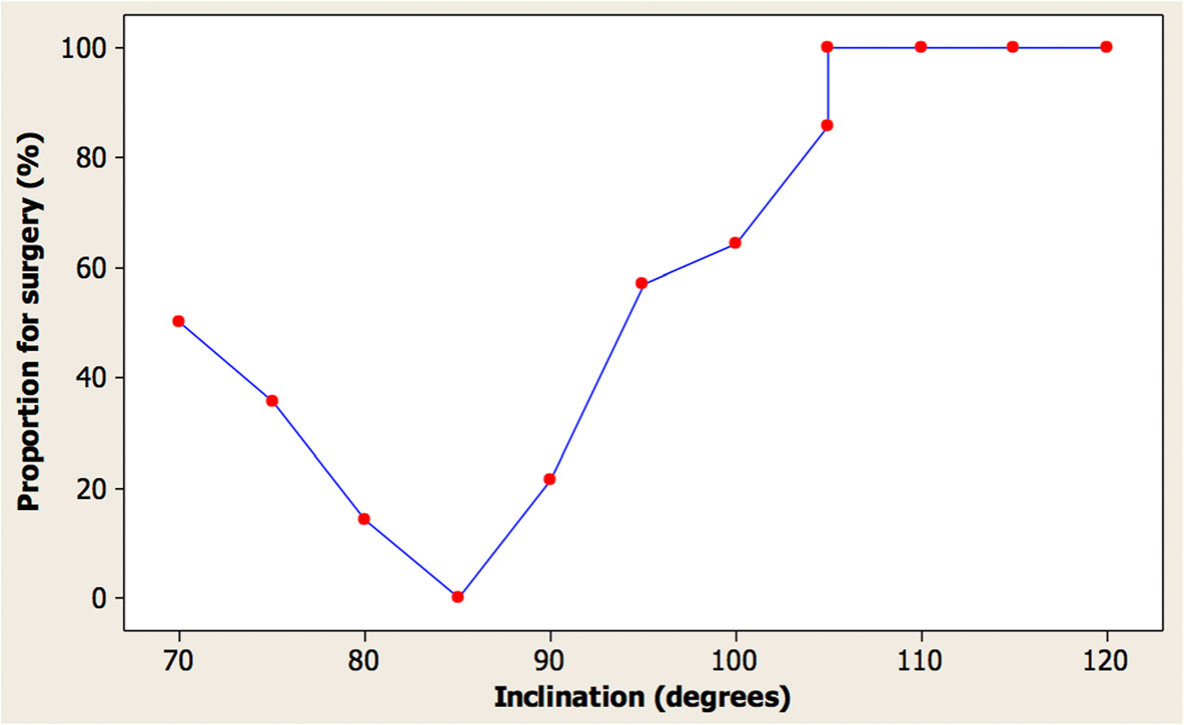
Proportion of clinicians suggesting a desire for treatment vs. inclination in female images

**Fig. 7:**
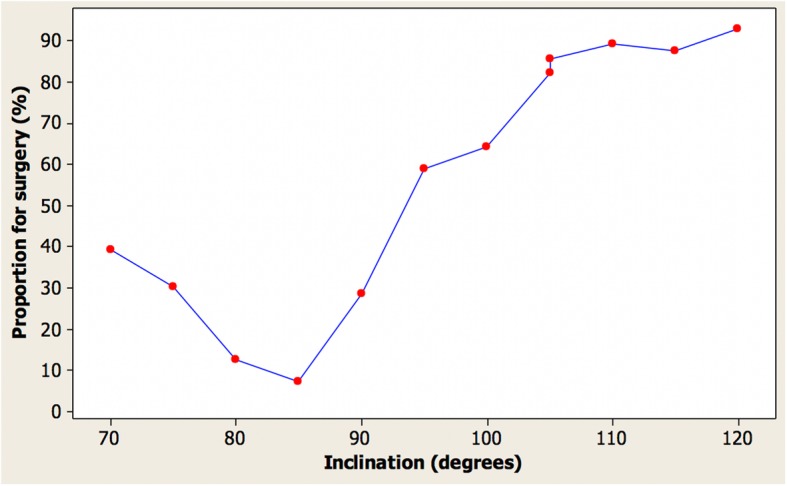
Proportion of laypeople suggesting a desire for treatment vs. inclination in female images

Figures 6 and 7 and Tables 3 and 4 indicate very good agreement in the desire for treatment between the groups of clinicians and laypersons. Repeatability was again excellent with very similar proportions suggesting a desire for treatment with the least good being the clinicians’ assessment of the images where one image had 86% and the other 100% suggesting a desire for treatment. Tables 3 and 4 show that the 85° inclination has the least desire for treatment for the images.

**Table 3:** Proportion of clinicians and laypeople suggesting a desire for treatment for images

Inclination (°)	Dentists (%)	Laypersons (%)
70	50	39
75	36	30
80	14	13
85	0	7
90	21	29
95	57	59
100	64	64
105	86	82
105	100	86
110	100	89
115	100	88
120	100	93

**Table 4:** Proportion of clinicians and laypeople suggesting a desire for treatment for images (ordered by proportion)

Inclination (°)	Dentists (%)	Laypersons (%)
85	0	7
80	14	13
90	21	29
75	36	30
70	50	39
95	57	59
100	64	64
105	86	82
105	100	86
115	100	88
110	100	89
120	100	93

## Discussion

The results of this investigation appear to indicate that with a patient in natural head position, the ideal inclination of a tangent to the labial face of the maxillary incisor crowns in profile view will be approximately parallel to the true vertical line and thereby approximately perpendicular to the true horizontal line. The most attractive inclination was the 85° retroclined position, with a range of 80 to 95° being acceptable in terms of observer acceptance. Overall, excessive proclination appears to be less desirable than retroclination, and beyond 105° most observers recommend treatment.

Cao et al. [11] and Chirivella et al. [12] evaluated profile smile attractiveness in relation to maxillary incisor inclination and sagittal position. Both groups of investigators used the Andrews’ method of assessing maxillary incisor position in relation to the inclination of the forehead [13], which is a questionable concept in relation to why the forehead inclination should be aesthetically relevant to incisor position, particularly considering the wide variation in forehead inclination within any ethnic population [3]. Nevertheless, Cao et al. [11] found that the most attractive image had a “5° lingual inclination,” which is approximately equivalent to the 85° inclination found in this investigation. Chirivella et al. [12] appear to suggest that the degree of proclination or retroclination depends on the facial type, with dolichocephalic facial patterns having maxillary incisor inclinations of 15° proclination or retroclination as the most attractive images. These results are not verified by the current investigation.

Giron de Velasco et al. [14] assessed the influence of “maxillary incisor torque,” by which they appear to mean incisor inclination in relation to the functional occlusal plane, on the aesthetic perception of the smile. Their online survey assessed three groups, and they found that laypeople preferred the 80° inclination, specialists in dental aesthetics preferred the 75° inclination, and the orthodontist group preferred the 70° inclination. Although the angles are not directly comparable as the authors were using different measuring parameters, nevertheless their results were significantly at variance with those of this investigation.

There are a number of practical implications for the results of this investigation. Orthodontists routinely alter the inclination of the maxillary incisors, and any major restoration of the maxillary anterior segment, from crowns and bridges to dental implants, must consider the inclination of the maxillary incisor crown face. The planned position of the maxillary incisor should be based on the position providing the best aesthetic result, i.e. the inclination of the labial face of the maxillary central incisor crowns in relation to the face, rather than on cephalometric values relating the entire long axis of the teeth to any anatomical reference plane. Additionally, treatment for orthognathic surgery patients with a significant anterior open bite often entails a differential posterior impaction of the maxilla, i.e. the posterior maxilla is elevated more than the anterior maxilla, with the maxilla rotating clockwise around the transverse axis, allowing the mandible to autorotate forward. Associated with such differential maxillary impaction, the maxillary incisors will retrocline, and, as such, a compensatory degree of incisor proclination must be built into the preoperative orthodontic preparation [15]. Additionally, if a segmental maxillary procedure is undertaken, rotation of the anterior segment containing the four incisor teeth will also change the inclination of the incisors [16]. In all these situations, the final inclination of the maxillary incisor crowns should be planned.

A purely anecdotal observation is the presence of a very mild Class II division 2 malocclusion, and thereby very mildly retroclined maxillary incisors, is rather common in attractive professional models and actors. Any relevance to the results of this investigation remains purely speculative.

## Conclusion

- The most attractive inclination of a tangent to the labial face of the maxillary incisor crowns in profile view in relation to the true horizontal line was 85°, i.e. 5° retroclined from a perpendicular 90° inclination.
- The most attractive range appears to be between 80 and 90°.
- Excessive proclination appears to be less desirable than retroclination.
- Beyond 105° proclination most observers recommend treatment.
- The profile smiling view is very useful for evaluation of the inclination of the labial face tangent and should be considered a standard view for orthodontic and orthognathic photographic records.

## Data Availability

Please contact the author for data requests.
